# Quantitative sonographic assessment of muscle thickness and fasciculations distribution is a sensitive tool for neuromuscular disorders

**DOI:** 10.1371/journal.pone.0292123

**Published:** 2023-09-28

**Authors:** Alon Abraham, Yaara Fainmesser, Vivian E. Drory, Vera Bril

**Affiliations:** 1 Neuromuscular Diseases Unit, Department of Neurology, Tel Aviv Sourasky Medical Center, Tel Aviv, Israel; 2 Sackler Faculty of Medicine, Tel-Aviv University, Tel-Aviv, Israel; 3 Ellen and Martin Prosserman Centre for Neuromuscular Diseases, Division of Neurology, Department of Medicine, University Health Network, University of Toronto, Toronto, Canada; Kaohsuing Medical University Hospital, TAIWAN

## Abstract

**Introduction:**

Loss of muscle thickness can be demonstrated in a wide spectrum of neuromuscular disorders, while fasciculations are more frequent in amyotrophic lateral sclerosis (ALS). In the current study, we aimed to determine the sensitivity and specificity of quantitative sonographic assessment of muscle thickness and the presence of fasciculations for diagnosing various neuromuscular disorders.

**Methods:**

The thickness and the presence of fasciculations in eight muscles were determined by sonography in patients with myopathy (22), polyneuropathy (36), ALS (91), and spinal muscular atrophy (SMA) (31) and compared to normative values determined in 65 heathy control subjects.

**Results:**

Reduced muscle thickness in at least one relaxed muscle showed 92–100% sensitivity for diagnosing a neuromuscular disease, with a specificity of 85% for differentiating patients from heathy controls (AUC = 0.90). Subtracting distal from proximal muscle thickness may differentiate between myopathy and polyneuropathy. Fasciculations in ≥1 proximal muscle showed good diagnostic accuracy (AUC = 0.87) for diagnosing ALS.

**Discussion:**

Sonographic assessment of muscle thickness is a sensitive tool for diagnosing a wide spectrum of neuromuscular diseases, and may facilitate diagnosis even in patients with normal strength on neurological examination, while the presence of fasciculations in proximal muscles may facilitate ALS diagnosis.

## Introduction

Neuromuscular ultrasound (US) is painless, non-invasive, and cost effective [1], and is being increasingly studied and used in neuromuscular clinics over the past decades. Muscle thickness loss can be demonstrated in a wide spectrum of neuromuscular disorders and is correlated with clinical and electrophysiological findings [2–6]. In addition, previous studies using neuromuscular US have demonstrated the presence of an increased number of fasciculations in neuromuscular diseases, most prominently in amyotrophic lateral sclerosis (ALS) [7–9]. Normal values for muscle thickness stratified by gender were previously determined, as well as the frequency of fasciculations in healthy controls [10]. Subsequently, normal values for muscle thickness were further stratified by age, and normal values for contracted muscle thickness were determined as well [11].

We hypothesized that neuromuscular US may be used as a point of care study complementary to the neurological examination, providing an objective biomarker for diagnosing and following patients with neuromuscular disorders, with a potential to increase the sensitivity and the accuracy of the neurological diagnosis. In order to support this assumption, we aimed to determine in this current proof of concept study, the sensitivity and specificity of quantitative sonographic assessment of muscle thickness and the presence of fasciculations for diagnosing various neuromuscular disorders, using our previously determined normal values [10, 11].

## Methods

The current study included heathy controls, as well as patients with myopathy, polyneuropathy, ALS, and spinal muscular atrophy (SMA). Patients with myopathy or polyneuropathy had compatible clinical presentation, confirmed by ancillary tests (e.g. electrophysiology, muscle biopsy, etc.). All patients with ALS fulfilled the revised El Escorial criteria for definite or probable ALS [12], and all patients with SMA had genetic confirmation. Healthy controls were prospectively recruited from the Prosserman Family Neuromuscular clinic, Toronto General Hospital, University Health Network, from October to December 2018. The Research Ethics Board of the University Health Network approved the study protocol and all subjects signed informed consent [10]. All patients were recruited subsequently at the neuromuscular diseases clinic at Tel Aviv Sourasky Medical Center, Tel Aviv, Israel, from December 2018 to December 2020. The Institutional Review Board of Tel Aviv Sourasky Medical Center approved the study protocol and all subjects signed an informed consent. All patients underwent routine clinical assessment, including muscle strength testing graded using the Medical Research Council (MRC) score [13]. Ten proximal and distal limb muscle groups were tested in all patients, including shoulder abduction, elbow flexion and extension, finger abduction and extension, hip flexion, thigh extension, ankle dorsiflexion and plantarflexion, and big toe extension, except for big toe extension in patients with SMA. MRC scores were recorded in the right limbs, unless symptoms were more pronounced on the left. Additional clinical scales included the ALS Functional Rating Scale Revised (ALSFRS-R) [14] in patients with ALS, and the Hammersmith Functional Rating Scale Expanded (HFMSE) [15] in patients with SMA.

All heathy controls and patients underwent quantitative sonographic assessment of muscle thickness and for the presence of fasciculations by a single examiner (AA) who was aware of their diagnoses, using the standardized Toronto protocol [10]. Healthy controls were assessed in the supine position using an ultrasound device (General Electric LOGIQ S7 Expert, Toronto, Canada), and a transducer with a frequency of 15 Hz (ML6-15), while patients were assessed in the supine position or seated in a wheelchair, using ultrasound device (Mindray M7, Shenzen, China), with a linear array transducer (L14-6Ns, Mindray, Shenzen, China). Muscle thickness was determined in eight relaxed muscles on the right side, unless symptoms were more pronounced on the left, including the biceps brachii (and the underlying brachialis muscle), abductor pollicis brevis (APB), first dorsal interosseous (FDI), abductor digiti minimi (ADM), quadriceps (including rectus femoris and vastus intermedius), tibialis anterior, extensor digitorum brevis (EDB), and abductor hallucis brevis (AHB), and is described elsewhere [10]. In addition, muscle thickness was assessed in 4 maximally contracted muscles (biceps brachii, APB, quadriceps, and tibialis anterior) [16]. Reduced relaxed or contracted muscle thickness was determined as lower than the 5^th^ percentile in healthy controls, stratified by age and gender [11]. Fasciculations were recorded during a time interval of 15 seconds, therefore detecting mainly muscles with a fasciculation frequency ≥ 4 per minute. In addition, we aimed to explore a potential novel sonographic marker for specificity, which reflects the relative loss of muscle thickness in proximal muscles compared with distal muscles: the biceps–EDB thickness, calculated by subtracting EDB muscle thickness from relaxed biceps muscle thickness (in cm) in each patient. We used a single proximal and distal muscle for simplicity and hypothesized that this value will be able to differentiate mainly between patients with myopathy and polyneuropathy. We chose the biceps muscle thickness and not the quadriceps, as the biceps is expected to be the least involved in polyneuropathy, and as the quadriceps is affected by age by threefold compared with the biceps controls [10]. The data that supports the findings of this study is available as a S1 Data.

### Statistical analysis

Data were analyzed using the statistical package for social sciences (SPSS) software version 27 (IBM Corp., Armonk, N.Y., USA). Comparisons of demographic data, muscle strength (using the MRC scale), and muscle thickness determined by US, between healthy controls and patients subgroups were performed using the Kruskal-Wallis test for continuous variables, or the χ2-test for categorical variables. The percent of subjects with reduced muscle thickness (below the 5^th^ percentile as determined in healthy controls), as well as the presence of at least one muscle with reduced thickness was calculated for healthy controls (for determining specificity) as well as for each patient subgroup (for determining sensitivity). Diagnostic accuracies were determined using the area under curve (AUC) of the receiver operating characteristic (ROC) curve. P values <0.05 were considered significant.

## Results

The study included 245 subjects: 65 heathy controls, as well as patients with myopathy (22), polyneuropathy (36), ALS (91), and SMA (31). The etiology for myopathy included idiopathic (6), facioscapulohumeral muscular dystrophy (FSHD) (4), limb-girdle muscular dystrophy (LGMD) (3), mitochondrial myopathy (3), inclusion body myositis (IBM) (2), necrotizing myopathy (2), hereditary inclusion body myopathy (1), and myotonic dystrophy type 2 (1). The etiology for polyneuropathy included chronic inflammatory demyelinating polyneuropathy (CIDP) (16), Charcot–Marie–Tooth disease (CMT) (8), idiopathic (4), diabetes (2), chemotherapy (2), prediabetes (1), anti-myelin associated glycoprotein (MAG) (1), systemic lupus erythematosus (SLE) (1), and small fiber neuropathy (1). Patients with ALS were the oldest with the shortest disease duration among patient subgroups, while patients with SMA were the youngest with the longest disease duration (Table 1). Muscle strength evaluation in patients using the MRC scale score and motor neuron disease scale scores is shown in Table 2. Patients with polyneuropathy had the lowest MRC score in big toe extension, and the highest MRC scores in proximal upper and lower limb muscles.

**Table 1:** Controls and patient subgroups’ characteristics.

	Controls	MYO	PNP	ALS	SMA	p-value
N	65	22	36	91	31	
Age (y)	47 ± 16	54 ± 18	52 ± 18	63 ± 13	34 ± 11	**<0.001**
Females (%)	63	59	58	44	58	0.16
BMI (kg/m^2^)	26 ± 5	25 ± 6	26 ± 5	25 ± 4	25 ± 7	0.71
Diabetes (%)	5	9	9	10	3	0.65
Disease Duration (y)		16 ± 1	7 ± 8	3 ± 2	30 ± 13	**<0.001**

MYO–Myopathy; PNP–Polyneuropathy; ALS–Amyotrophic Lateral Sclerosis; SMA–Spinal Muscular Atrophy. BMI—Body mass index. Data presented as mean ± standard deviation unless indicated otherwise. P-values for comparisons between patient subgroups. Significant p-values (<0.05) are bolded.

**Table 2:** Comparisons of MRC scores, and clinical scales between patient subgroups.

	MYO	PNP	ALS	SMA	p-value
N	22	36	91	31	
**MRC scores (0–5):**					
Shoulder Abduction	4.3 ± 0.8	4.9 ± 0.3	3.3 ± 1.8	3.3 ± 1.4	**<0.001**
Elbow Flexion	4.2 ± 0.8	5 ± 0.2	3.7 ± 1.7	3.8 ± 1	**<0.001**
Elbow Extension	4.3 ± 0.8	5 ± 0.2	3.9 ± 1.5	3.5 ± 1.2	**<0.001**
Finger Abduction	4.3 ± 0.6	3.8 ± 1.4	2.4 ± 1.9	3.5 ± 1.1	**<0.001**
Finger Extension	4.3 ± 0.6	4.2 ± 1.1	3.1 ± 1.6	3.6 ± 1	**<0.001**
Hip Flexion	3.5 ± 1.3	4.8 ± 0.5	3.5 ± 1.7	1.4 ± 0.9	**<0.001**
Knee Extension	4.2 ± 1.4	5 ± 0.2	4.1 ± 1.6	1.4 ± 1.2	**<0.001**
Dorsiflexion	3.4 ± 1.8	3.4 ± 2	3.2 ± 2.1	3.4 ± 1.5	0.91
Plantarflexion	4.4 ± 1.5	4.5 ± 1.4	4.2 ± 1.6	4.3 ± 1	0.16
Big Toe Extension	3.8 ± 1.9	2.9 ± 2.1	3.1 ± 2.1	-	0.17
Elbow Flexion–Big Toe Extension	0.5 ± 2	2.1 ± 2.1	0.8 ± 1.9		**0.002**
**ALSFRSR (0–48)**			30 ± 9		
**HAMMERSMITH (0–69)**				21 ±18	

MYO–Myopathy; PNP–Polyneuropathy; ALS–Amyotrophic Lateral Sclerosis; SMA–Spinal Muscular Atrophy; BB- Biceps brachii; FDI- First dorsal interosii; APB- Abductor pollicis brevis; ADM- Abductor digiti minimi; QUAD- Quadriceps; TA- Tibialis anterior; AH- Abductor hallucis; EDB- Extensor digitorum brevis; ALSFRS-R—ALS functional rating scale-revised. Data presented as mean ± standard deviation. P-values for comparisons between patient subgroups. Significant p-values (<0.05) are bolded.

### Muscle thickness

**Fig 1:**
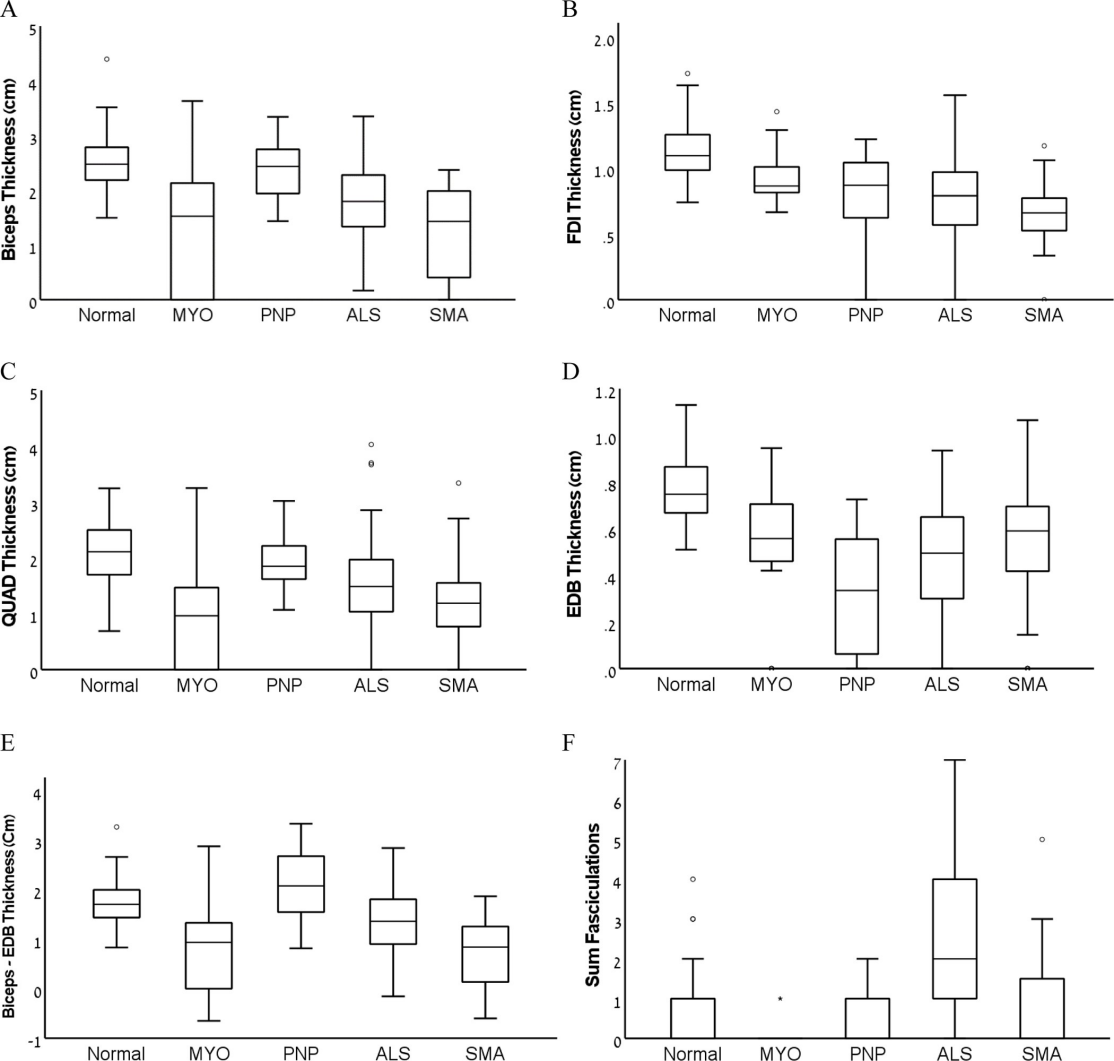
Comparisons of muscle thickness in selected muscles and the number of muscles showing fasciculations across healthy controls and patients with various neuromuscular disorders. MYO–Myopathy; PNP–Polyneuropathy; ALS–Amyotrophic Lateral Sclerosis; SMA–Spinal Muscular Atrophy FDI–First Dorsal Interosseous; QUAD–Quadriceps; EDB—Extensor Digitorum Brevis; Sum Fasciculations—Number of muscles showing fasciculations.

The results of quantitative sonographic assessment of muscle thickness are shown in Table 3. The lowest muscle thickness in the EDB muscle across all patient subgroups was found in patients with polyneuropathy, as well as the highest muscle thickness in the proximal upper and lower limb muscles. The lowest muscle thickness in proximal muscles was found in patients with myopathy and SMA. Biceps–EDB thickness value showed poor diagnostic accuracy (AUC = 0.67) for differentiating between patient groups and controls, but a good diagnostic accuracy for differentiating patients with polyneuropathy and myopathy (AUC = 0.83) due to significant overlap (Fig 1).

**Table 3:** Comparisons of muscle thickness and the number of muscles showing fasciculations between healthy controls and patient subgroups.

	Controls	MYO	PNP	ALS	SMA
N	65	22	36	91	31
**Relaxed (cm)**					
Biceps	2.51 ± 0.52	1.42 ± 1.10	2.35 ± 0.57	1.81 ± 0.70	1.25 ± 0.84
FDI	1.13 ± 0.20	0.94 ± 0.20	0.87 ± 0.55	0.75 ± 0.33	0.66 ± 0.23
APB	0.44 ± 0.08	0.37 ± 0.10	0.32 ± 0.11	0.26 ± 0.12	0.32 ± 0.13
ADM	0.97 ± 0.18	0.96 ± 0.18	0.93 ± 0.22	0.84 ± 0.25	0.69 ± 0.22
Quadriceps	2.13 ± 0.55	1.05 ± 0.92	1.89 ± 0.48	1.55 ± 0.75	1.23 ± 0.78
Tibialis Anterior	2.12 ± 0.32	1.08 ± 1.24	1.69 ± 0.93	1.52 ± 1.03	0.70 ± 0.82
AHB	1.19 ± 0.20	0.93 ± 0.18	0.83 ± 0.18	0.97 ± 0.17	0.95 ± 0.27
EDB	0.76 ± 0.14	0.54 ± 0.26	0.32 ± 0.25	0.46 ± 0.24	0.54 ± 0.29
Biceps–EDB	1.75 ± 0.46	0.88 ± 1.07	2.06 ± 0.63	1.35 ± 0.67	0.72 ± 0.73
**Contracted (cm)**					
Biceps	3.52 ± 0.65	2.31 ± 1.65	3.45 ± 0.46	2.77 ± 0.90	1.88 ± 1.26
APB	0.60 ± 0.12	0.49 ± 0.15	0.42 ± 0.16	0.35 ± 0.18	0.44 ± 0.17
Quadriceps	3.09 ± 0.70	1.58 ± 1.32	2.70 ± 0.60	2.20 ± 0.94	1.36 ± 0.86
Tibialis Anterior	2.68 ± 0.34	1.29 ± 1.48	1.99 ± 1.11	1.77 ± 1.19	0.79 ± 0.95
**Fasciculations (n)** *	0.91 ± 0.94	0.09 ± 0.29	0.41 ± 0.70	2.5 ± 1.65	0.94 ± 1.34
**Fasciculations (n)** **	0.02 ± 0.12	0	0.03 ± 0.17	0.97 ± 0.69	0.16 ± 0.37

MYO–Myopathy; PNP–Polyneuropathy; ALS–Amyotrophic Lateral Sclerosis; SMA–Spinal Muscular Atrophy; FDI–First Dorsal Interosseous; APB–Abductor Pollicis Brevis; ADM–Abductor Digiti Minimi; QUAD–Quadriceps; TA–Tibialis Anterior; AHB–Abductor Hallucis Brevis; EDB–Extensor Digitorum Brevis. Muscle thickness values are in cm.

Fasciculations*–Number of muscles showing fasciculations.

Fasciculations**—Number of muscles showing fasciculations including the biceps and quadriceps only. Data presented as mean ± standard deviation P-values were < 0.001 for all comparisons.

Table 4 shows the percent of muscles with reduced thickness in each group (reflecting test sensitivity), as well as in controls (reflecting 1-specificity), and the AUC for differentiating any neuromuscular disease from controls based on muscle thickness. Reduced muscle thickness using the 5^th^ percentile stratified by age and gender as determined by healthy controls in at least one relaxed muscle showed 92–100% sensitivity for diagnosing a neuromuscular disease, with a specificity of 85% for differentiating patients from healthy controls (AUC = 0.90). Reduced muscle thickness in at least one contracted muscle showed lower sensitivities, which was most prominent for polyneuropathy (56%), but with higher specificity of 94% (AUC = 0.87). For comparison purposes, we also calculated the MRC score of elbow flexion–big toe extension for differentiating between patient subgroups (Table 2). This score showed only fair diagnostic accuracy for differentiating between patients with polyneuropathy and myopathy (AUC = 0.74).

**Table 4:** Sensitivities in percentages and area under curve (AUC) for quantitative sonographic assessment of muscle thickness and the presence of fasciculations for diagnosing a neuromuscular disease.

	Controls	MYO	PNP	ALS	SMA	AUC
N	65	22	36	91	31	245
**Relaxed (%)**						
Biceps	2	73	19	51	81	0.78
FDI	2	36	44	57	77	0.84
APB	2	50	56	78	61	0.85
ADM	2	9	22	34	74	0.67
Quadriceps	2	64	14	31	74	0.76
Tibialis Anterior	2	55	33	41	87	0.70
AHB	2	41	50	26	45	0.80
EDB	5	45	81	67	48	0.85
***Overall Sensitivity (%)***	***15***	***100***	***92***	***93***	***100***	***0*.*90***
**Contracted (%)**						
Biceps	2	50	17	58	77	0.75
APB	2	27	36	63	29	0.83
Quadriceps	2	59	11	36	84	0.80
Tibialis Anterior	3	55	42	46	90	0.78
***Overall Sensitivity (%)***	***6***	***91***	***56***	***82***	***97***	***0*.*87***
Fasciculations* *(%)*	27	0	12	70	28	0.75
Fasciculations** *(%)*	2	0	3	78	16	0.87

MYO–Myopathy; PNP–Polyneuropathy; ALS–Amyotrophic Lateral Sclerosis; SMA–Spinal Muscular Atrophy;; FDI–First Dorsal Interosseous; APB–Abductor Pollicis Brevis; ADM–Abductor Digiti Minimi; QUAD–Quadriceps; TA–Tibialis Anterior; AHB–Abductor Hallucis Brevis; EDB–Extensor Digitorum Brevis.

Fasciculations*– ≥2 fasciculations in 8 studied muscles

Fasciculations**– ≥1 fasciculation in biceps and quadriceps only; Sensitivities for controls reflects false positive rates. AUC was calculated for differentiating any neuromuscular disease from controls based on muscle thickness, and for diagnosing ALS based on fasciculations.

Within patients with normal strength on neurological examination (11 with polyneuropathy, 5 with ALS and 1 with myopathy), most (73–100%) demonstrated reduced muscle thickness (Table 5).

**Table 5:** Sensitivities in percentages for quantitative sonographic assessment of muscle thickness and the presence of fasciculations for diagnosing a neuromuscular disease in patients with normal strength on neurological examination.

	Controls	MYO	PNP	ALS
N	65	1	11	5
**Relaxed (%)**				
Biceps	2	0	27	0
FDI	2	0	0	0
APB	2	0	36	0
ADM	2	0	0	0
Quadriceps	2	100	9	
Tibialis Anterior	2	0	9	60
AHB	2	0	64	20
EDB	5	0	55	40
***Overall Sensitivity (%)***	***15***	***100***	***73***	***80***
**Contracted (%)**				
Biceps	2	0	18	0
APB	2	0	18	0
Quadriceps	2	100	9	0
Tibialis Anterior	3	0	18	60
***Overall Sensitivity (%)***	***6***	***100***	***27***	***60***
Fasciculations* *(%)*	27	0	24	60
Fasciculations** *(%)*	2	0	0	60

MYO–Myopathy; PNP–Polyneuropathy; ALS–Amyotrophic Lateral Sclerosis; SMA–Spinal Muscular Atrophy;; FDI—First Dorsal Interosseous; APB—Abductor Pollicis Brevis; ADM—Abductor Digiti Minimi; QUAD–Quadriceps; TA–Tibialis Anterior; AHB–Abductor Hallucis Brevis; EDB—Extensor Digitorum Brevis.

Fasciculations*– ≥2 fasciculations in 8 studied muscles

Fasciculations**– ≥1 fasciculation in biceps and quadriceps only; Sensitivities for controls reflects false positive rates.

### Fasciculations

Fasciculations in proximal and distal muscles were most frequent in patients with ALS, but were also found in other groups, including healthy subjects (Fig 1). However, use of proximal muscles (biceps and quadriceps) only for determining fasciculation frequency was more specific, showing significantly lower frequencies of fasciculations, especially in healthy controls (Table 3). Diagnostic accuracy was determined considering the number of proximal and distal muscles with two or more fasciculations and showed relatively low specificity in ALS patients compared with controls (73%), with only fair diagnostic accuracy (AUC = 0.75). However, using only proximal muscles (biceps and quadriceps) showed significantly higher specificity (98%) compared with controls and good diagnostic accuracy (AUC = 0.87) for differentiating ALS from controls and other patient subgroups.

Fasciculations in proximal muscles were demonstrated in 3 out of 5 patients with ALS and normal strength on neurological examination, (Table 5).

## Discussion

In the last decades, neuromuscular US has been increasingly used in clinic and research, and has been proposed as a complementary test in the electrophysiology laboratory. Several important advantages of US include being an accurate point of care tool, which is painless, performed quickly and safely, and providing dynamic and structural information. The accuracy of quantitative sonographic assessment of muscle thickness for diagnosing various neuromuscular disorders has not been adequately investigated. The current study indicates that quantitative sonographic assessment of muscle thickness using a standardized protocol imaging of 8 relaxed muscles [10], and normative values stratified by age and sex, has excellent diagnostic accuracy (AUC = 0.90; sensitivities: 92–100%, specificity: 85%) for differentiating patients with various neuromuscular disorders from controls. Furthermore, most patients (12 out of 17) demonstrating normal strength on neurological examination including commonly tested muscles, show abnormal low muscle thickness in at least one muscle, suggesting that neuromuscular US may facilitate diagnosis, although numbers are relatively low in order to conclude a definite conclusion.

In contrast, examining four contracted muscles showed inferior diagnostic accuracy, with reduced sensitivity mainly for diagnosing polyneuropathy (sensitivity: 56%), most likely explained by the lower number of muscles studied in the contracted state and the lack of inclusion of distal lower limb muscles which are the most commonly involved in polyneuropathy. However, muscle selection studied in the contracted state was determined based on feasibility, which seemed by the authors to be higher in proximal muscles.

In order to explore a potential sonographic marker for specificity, which reflects the relative loss of muscle thickness in proximal muscles compared with distal muscles, we calculated the biceps–EDB thickness value. Although this novel value showed poor diagnostic accuracy (AUC = 0.67) for differentiating between patient subgroups and controls, good diagnostic accuracy was demonstrated (AUC = 0.83) for differentiating between patients with polyneuropathy and myopathy, reflecting the preferential proximal muscle loss in myopathy, and distal muscle loss in polyneuropathy. This value also showed relative preferential proximal muscle loss in SMA as expected [17], and preferential distal muscle loss in ALS, as commonly seen in clinic, and possibly explaining the higher yield of EMG sampling of distal muscles in ALS [18]. However, in contrast to the more pronounced differences between myopathy and polyneuropathy, differentiating between different motor neuron diseases and between motor neuron diseases and other neuromuscular disorders using this value is limited due to significant overlap (Fig 1).

Although we found that fasciculations were most frequent in patients with ALS, observed in at least two muscles in 70% of patients, they were not uncommon in other neuromuscular disorders except myopathy and were even demonstrated in at least two muscles in 27% of healthy controls, therefore limiting their diagnostic accuracy (AUC = 0.75). These results show a lower diagnostic accuracy when compared with other studies using two or more muscles with fasciculations to diagnose ALS [7–9]. The high rate of fasciculations in healthy controls in our cohort which contributed to lower specificity might be related to muscle selection in our study, such as the FDI and AHB, which demonstrate fasciculations frequently, and were not included in other studies [8, 9]. In addition, the lower sensitivity for diagnosing ALS in our study might be related to the smaller number of muscles examined (11 vs. 11–21 muscles [7–9]), and the shorter scan time for detecting fasciculations (15 vs. 30–60 seconds [7–9]). Nonetheless, as previously reported [9], fasciculations in proximal muscles were more sensitive and specific for an ALS diagnosis, and were found in our cohort in at least one muscle in 78% of patients with ALS, as compared to only 2% of healthy controls, with good accuracy for differentiating between ALS and other diagnoses (AUC = 0.87).

Our study has several limitations. Healthy subjects and patients underwent sonographic muscle examination by a single examiner (AA), who was aware of their diagnosis, and with different US devices. However, muscle thickness is not expected to be influenced by device type, in contrast to echogenicity. In addition, patients with myopathy and polyneuropathy were chosen as a convenience sample and are therefore not representative of a community or even a tertiary clinic. For example, the leading etiologies for polyneuropathy in our cohort were CIDP and CMT, which might present with more prominent muscle atrophy compared with the most common etiology for polyneuropathy which is diabetes [19]. Consequently, sonographic assessment of muscle thickness may be less sensitive in the more common etiologies for myopathy and polyneuropathy. However, this study was designed as a proof-of-concept study, and further studies are warranted in order to explore sensitivities for various etiologies. Finally, we have not explored additional sonographic muscle characteristics such as echogenicity, elastography or fat distribution.

In conclusion, quantitative sonographic assessment of muscle thickness is a promising tool which is highly sensitive in a wide spectrum of neuromuscular disorders, may facilitate diagnosis even in patients with normal strength on neurological examination.

In addition, sonographic evaluation of fasciculations limited to proximal muscles has good accuracy for diagnosing ALS and is superior to examining proximal and distal muscles. Further studies are required to confirm these findings, and for evaluating broader etiologies for neuromuscular disorders.

## Supporting information

S1 Data (XLSX)
